# Revolutionizing Diabetes Management Through Nanotechnology-Driven Smart Systems

**DOI:** 10.3390/pharmaceutics17060777

**Published:** 2025-06-13

**Authors:** Aayush Kaushal, Aanchal Musafir, Gourav Sharma, Shital Rani, Rajat Kumar Singh, Akhilesh Kumar, Sanjay Kumar Bhadada, Ravi Pratap Barnwal, Gurpal Singh

**Affiliations:** 1 University Institute of Pharmaceutical Sciences, Panjab University, Chandigarh 160014, India; aayushkaushal662@gmail.com (A.K.); aanchalmusafir22@gmail.com (A.M.); gouravpandit2140@gmail.com (G.S.); 2 Department of Biophysics, Panjab University, Chandigarh 160014, India; siwachshital1998@gmail.com (S.R.); singhrajat0702@gmail.com (R.K.S.); 3 Division of Medicine, ICAR-Indian Veterinary Research Institute, Izatnagar, Bareilly 243122, India; dr_akhil2005@yahoo.co.in; 4 Department of Endocrinology, PGIMER, Chandigarh 160012, India; bhadadask@rediffmail.com

**Keywords:** diabetes mellitus, nanotechnology, nanomedicine, insulin delivery, glucose monitoring, smart nanocarriers, continuous glucose monitoring (CGM), microneedles, carbon nanomaterials, quantum dots (QDs), polymeric nanoparticles, wearable biosensors, non-invasive monitoring, AI in diabetes management, glucose biosensors, drug delivery systems, electrochemical biosensors, nanofibers, diabetic wound healing

## Abstract

Diabetes is a global health challenge, and while current treatments offer relief, they often fall short in achieving optimal control and long-term outcomes. Nanotechnology offers a groundbreaking approach to diabetes management by leveraging materials at the nanoscale to improve drug delivery, glucose monitoring, and therapeutic precision. Early advancements focused on enhancing insulin delivery through smart nanosystems such as tiny capsules that gradually release insulin, helping prevent dangerous drops in blood sugar. Simultaneously, the development of nanosensors has revolutionised glucose monitoring, offering real-time, continuous data that empowers individuals to manage their condition more effectively. Beyond insulin delivery and monitoring, nanotechnology enables targeted drug delivery systems that allow therapeutic agents to reach specific tissues, boosting efficacy while minimising side effects. Tools like microneedles, carbon nanomaterials, and quantum dots have made treatment less invasive and more patient-friendly. The integration of artificial intelligence (AI) with nanotechnology marks a new frontier in personalised care. AI algorithms can analyse individual patient data to adjust insulin doses and predict glucose fluctuations, paving the way for more responsive, customised treatment plans. As these technologies advance, safety remains a key concern. Rigorous research is underway to ensure the biocompatibility and long-term safety of these novel materials. The future of diabetes care lies in the convergence of nanotechnology and AI, offering personalised, data-driven strategies that address the limitations of conventional approaches. This review explores current progress, persistent challenges, and the transformative potential of nanotechnology in reshaping diabetes diagnosis and treatment and improving patient quality of life.

## 1. Introduction

**Figure 1:**
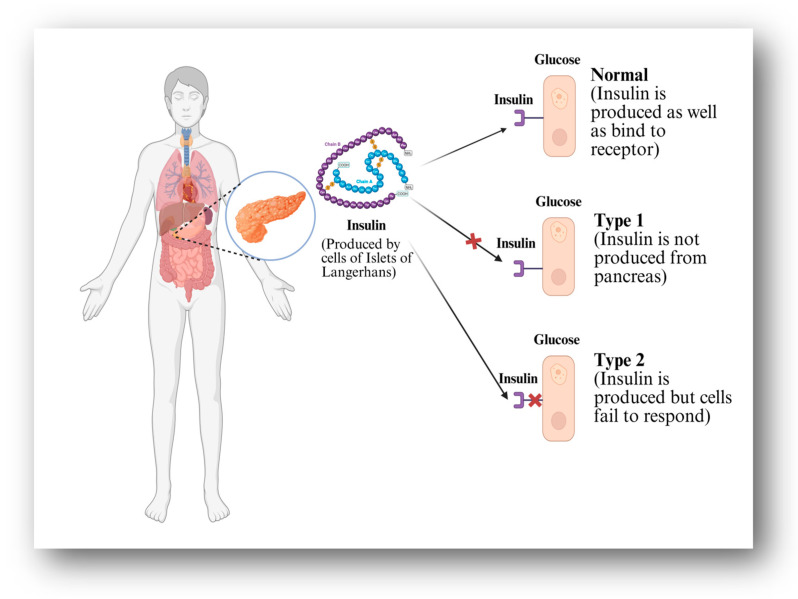
Mechanism and classification of diabetes based on insulin production and response.

Diabetes mellitus, a kind of endocrine malfunction, is one of the most frequent medical problems today. This condition is characterised by elevated blood sugar levels. The underlying reason for this indisposition might be the malfunction of pancreatic beta cells, or it could be the result of an inefficient response to the insulin that is produced. The three most common symptoms of diabetes mellitus are polyuria (frequent urination), polydipsia (excessive thirst), and polyphagia. Diabetes is often classified into three types: People with Type 1 diabetes, also called insulin-dependent diabetes mellitus (IDDM), cannot make enough insulin. People with Type 2 diabetes, also called non-insulin-dependent diabetes mellitus (NIDDM), can use insulin, but their cells are not sensitive to it. And finally, there is gestational diabetes, which is also known as diabetes during pregnancy. Type 2 diabetes is often referred to as adult-onset diabetes, while type 1 diabetes is typically referred to as juvenile diabetes [1]. Type 1 diabetes mellitus (T1DM) is a chronic autoimmune disease that produces hyperglycemia owing to insulin deficiency induced by pancreatic islet cell destruction [2]. Type 2 diabetes is induced by insulin resistance in cells [3]. The causes and types of diabetes are depicted in Figure 1. Globally, the number of people with diabetes is increasing at epidemic levels. Diabetes and related disorders account for a significant amount of annual healthcare costs. A variety of risk factors impact the disease’s origin and progression to epidemic status. Although there is no known cure for diabetes, it appears to be manageable by regular self-care, effective diabetes education, and significant improvements in management, knowledge, attitudes, and abilities [4]. The major causes of the increased type 2 diabetes prevalence include population ageing, an increase in obesity prevalence worldwide, and unhealthy lifestyle choices such as poor diets and inactivity, all of which are compounded by larger socioeconomic factors such as urbanisation [5]. Diabetes affects around 537 million people aged 20 to 79 worldwide (or roughly 10.5% of individuals in this age group). Diabetes is estimated to affect 643 million people globally by 2030, rising to 783 million by 2045 [6]. Diabetes caused 2.5% of all fatalities globally in 2017, totalling around 1.4 million deaths. The prevalence of diabetes in low- and middle-income nations indicates that the epidemiology of diabetes varies between high-income and low-income countries [7].

NIDDM, or adult-onset diabetes, is the most prevalent kind of diabetes, accounting for around 85 out of every 100 cases. Both types of diabetes can cause a range of complications that affect numerous bodily systems. These problems might manifest as macrovascular endpoints like peripheral vascular disease, ischaemic heart disease, and stroke, or microvascular endpoints like retinopathy, nephropathy, and neuropathy. Diabetes is a critical public health problem due to its association with early morbidity, mortality, reduced life expectancy, and significant financial and societal consequences. This study concludes that diabetes mellitus has emerged as a major global health issue due to its rising prevalence. If this issue remains unaddressed, it may present more issues for communities in the future. As a result, further diabetes preventive and management activities are required. Diabetes mellitus can aggravate and complicate other disorders, create difficulties in many organs and tissues, and result in severe financial obligations. According to estimates, persons with diabetes mellitus spend 2.3 times more on routine healthcare than nondiabetic individuals [8].

In both industrialised and emerging nations, the prevalence of obesity and diabetes is notably rising. Communicable diseases have taken precedence over the growing battle with non-communicable diseases. The United Nations General Assembly (UNGA) recognised diabetes as a global public health issue in 2006 and advised member states to put policies in place to reduce its effects. Diabetes-related medical expenses are high and are constantly rising. An estimated USD 727 billion was spent on diabetes-related medical care worldwide in 2017. By 2045, this expense is predicted to have increased to $776 billion from USD 232 billion in 2006 (International Diabetes Federation, 2017) [9]. Apart from the conventional vascular and neuropathic consequences associated with diabetes, physical and mental impairments are just now becoming recognised as significant types of complications in people with diabetes that disproportionately impact older adults. The pathway to physical handicap and lower-limb dysfunction is mediated by the combination of sarcopenia and frailty, which is frequently made worse by different forms of neuropathy [10]. Conversely, frequent episodes of hypoglycaemia and chronic hyperglycaemia double the risk of cognitive impairment and all forms of dementia [11]. Additionally, diabetes raises the chance of incident depression by 27% [12].

Antidiabetic medication development is not without its difficulties. Nonetheless, a strong drug development procedure with strict “Go–No Go” judgements will help to end programmes early or introduce better medications to the market. Future medications for type 2 diabetes are probably going to be novel medications with many sites of action that positively target several aspects of the metabolic syndrome. The insulin of the future for both type 1 and type 2 diabetes is probably going to be an insulin analogue that mimics physiological insulin production and is easy to administer in cutting-edge delivery systems [13].

In this review, we have explored the growing burden of diabetes mellitus worldwide, especially its increasing prevalence in both developed and developing countries. We have discussed the different types of diabetes and their underlying causes, complications, and the challenges they pose to healthcare systems. The review also covered current antidiabetic medications, their mechanisms, and limitations. Furthermore, we highlighted the urgent need for improved therapies, including the development of safer and more effective drugs and delivery systems. Overall, this review highlights the importance of innovative approaches such as nanotechnology to better manage and treat diabetes and reduce its long-term health and economic impacts.

## 2. Conventional Antidiabetic Regimens: Mechanisms, Efficacy, and Modern-Day Relevance

Diabetes is a prevalent disease in the Indian population, particularly among those who are overweight, obese, or inactive. The primary characteristic of diabetes is persistently elevated blood sugar levels, which are caused by a combination of lifestyle, environmental, and genetic factors that result in insulin resistance and issues with insulin production. This dysfunctional condition affects the delicate balance of glucose regulation, leading to issues in both large and small blood vessels, significantly affecting the rates of sickness and mortality. New blood glucose-lowering drugs have been developed over the past decade, and diabetes raises the risk of additional co-morbidities such as fractures, cardiovascular problems, and several types of cancer [14]. Oral hypoglycaemic agents that help increase insulin secretion include sulfonylureas, which lower blood sugar levels by blocking KATP channels and stimulating beta cells in the pancreas to produce more insulin. However, sulfonylureas can cause weight gain and hypoglycaemia, while insulin or sulfonylureas have reduced the incidence of microvascular problems [15,16]. Meglitinides/Phenylalanine analogues are insulin secretagogues called glides (meglitinides) that depolarize pancreatic cells, increasing the release of insulin. These drugs have a short duration of action and are rapid-acting [17,18,19]. Metformin is the first medicine usually given to overcome insulin resistance in type 2 diabetes (T2DM), which belongs to the biguanide class of drugs. Bi-guanides do not release insulin, but their activity depends on the presence of insulin [20]. The activation of AMP-activated protein kinase (AMPK) is essential in facilitating the effects of metformin, which inhibits hepatic gluconeogenesis and the liver’s glucose output, improving insulin-mediated glucose absorption and its retention in skeletal muscle and fat [21]. Thiazolidinediones (TZDs) act by binding to a nuclear receptor called peroxisome proliferator-activated receptor gamma (PPAR-γ), helping regulate genes that control glucose and fat metabolism, improving insulin sensitivity [22]. Miscellaneous drugs like α-glucosidase inhibitors work by slowing down the action of the enzyme α-glucosidase in the upper part of the intestine, which normally breaks down complex sugars into simple sugars. SGLT-2 inhibitors lower blood glucose levels by blocking the SGLT-2 proteins in the proximal renal tubules, reducing glucose reabsorption into the blood. They are a safer alternative to other diabetes treatments since they are less likely to cause hypoglycaemia, particularly when taken with other antidiabetic medications. Among the drug classes discussed, GLP-1 (glucagon-like peptide 1) agonists and SGLT-2 (Sodium–glucose co-transporter 2) inhibitors show benefits in reducing case mortality and cardiac events. SGLT-2 inhibitors are the choice of drug for patients suffering from end-stage kidney disease. However, their effects were deemed insignificant since they failed to meet the criteria for minimally significant changes. TZD can preserve beta cell function of the pancreas, lower blood sugar levels, and sometimes have beneficial effects on lipid profiles and cardiovascular health [23,24]. Nevertheless, numerous conventional medications are associated with adverse effects, including hypoglycaemia, weight gain, and limited long-term safety, despite their efficacy. These limitations highlight the necessity of safer, more targeted approaches, such as nanotechnology-based therapies, which have the potential to improve patient outcomes, reduce adverse effects, and enhance drug delivery in diabetes management. As we know, nanotechnology-based therapies have shown the ability to directly deliver insulin to target cells and thereby reduce the risk of hypoglycaemia and weight gain that are associated with conventional treatments. Table 1 provides an overview of these drugs, including standard dosage ranges, interactions with food intake, and often reported adverse effects, for clarity and comparison purposes.

## 3. Nanoscale Innovations in Diabetes Care

**Figure 2:**
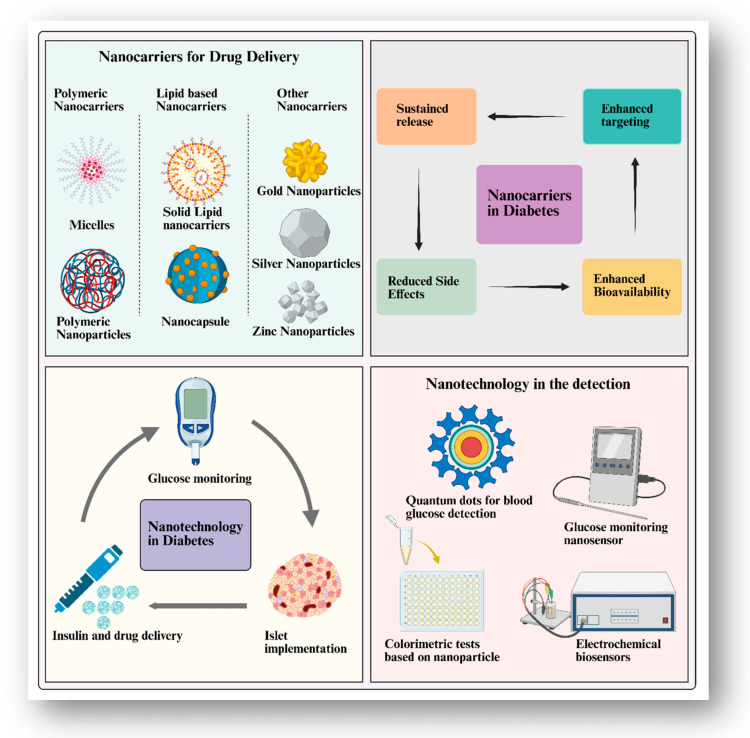
Overview of pharmaceutical nanosystems in diabetes, illustrating nanocarriers for targeted drug delivery and nanosensors for glucose monitoring and diagnosis.

Nanotechnology refers to the confluence of science, technology, and engineering at the nanoscale. In essence, this scale runs from 1 to 100 nm. As a result, to create new large-scale materials, researchers are using exceedingly tiny particles. More specifically, nanotechnology is the controlled manipulation of size and shape at the nm scale for imaging, modelling, measuring, creating, characterising, manufacturing, and applying structures, devices, and systems [24,35]. With advances in materials science, chemistry, and engineering over the last few decades, nanotechnology is currently being applied in a range of novel applications. Various pharmacological nanosystems have been depicted in Figure 2.

**Figure 3:**
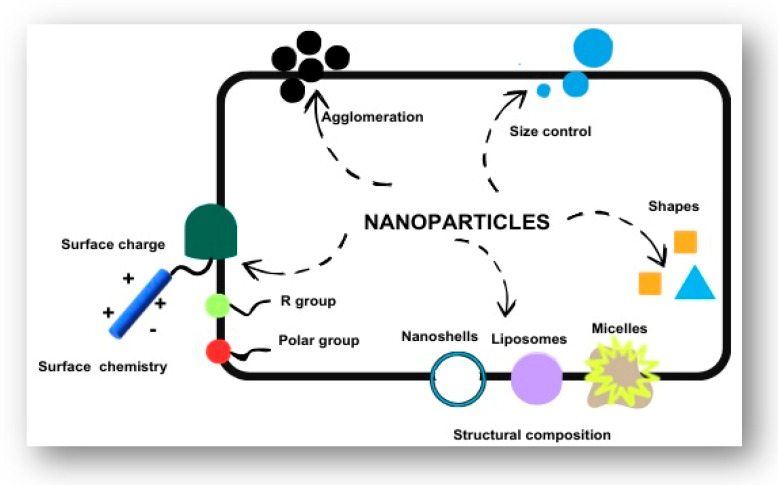
Schematic showing variations in nanoparticle properties such as surface charge, size, shape, and structure affecting their functionality.

Nanomedicine is a prominent field that provides precise techniques for illness prevention, diagnosis, and treatment. For example, nanoparticles can help to carry medicine directly to target cells, which is useful in cancer therapy. Furthermore, pharmaceuticals and active molecules may be altered at the nanoscale to regulate and modify essential properties, resulting in increased solubility, controlled release, and targeted drug delivery [36]. Nanotechnology contributes to tissue engineering by helping to repair or recreate damaged tissues. Furthermore, many nanotechnology-based sensors can now detect chemical and biological pollutants in water, air, and soil with substantially better sensitivity, which helps to minimise environmental pollution [24]. This allows for more accurate and precise environmental monitoring. Beyond healthcare, nanotechnology is important in infrastructure and has several uses in a variety of industries, including food, cosmetics, and diagnostics [35]. Focusing on its historical development in diabetes treatment, the journey began in 1962 when Clark and Lyons created the first biosensor employing electrochemical transductions linked with enzymes by inserting glucose oxidase (GOx) in a solution between a membrane and an electrode. Since then, various biosensors have been created, most of which are based on two concepts [37]. The first category is electrochemical biosensors, which use electrochemical transduction enzymes like GOx or glucose dehydrogenase (GDH) [38]. Initially, these sensors functioned by entrapping GOx within polymers or membranes put on a metal or carbon working electrode, which served as the transducer and was linked to an electron mediator. Electrochemical species emitted during enzymatic activity were detected on the electrode surface. However, the primary obstacles were interference from endogenous species such as uric acid and ascorbic acid, as well as the requirement for high working potentials. To solve these difficulties, second-generation glucose biosensors substituted oxygen with redox mediators like ferrocene, which aid in electron transport from the enzyme to the electrode surface. The mediator would be reduced and reoxidised at the electrode, generating an amperometric signal and replenishing the oxidised mediator, enhancing selectivity and making it appropriate for implanted blood glucose monitoring systems [39]. The second kind is optical biosensors, which employ binding proteins, receptors, or inactive apo-enzymes to design reversible, implantable, and inline sensing devices. Veetil et al. [39] created a glucose sensor protein that generates FRET signals to monitor glucose levels from 25 to 800 μM. Nanotechnology has enabled the continual development of better glucose monitoring and insulin delivery procedures, considerably increasing the quality of life for people with diabetes. Innovations include glucose-responsive insulin delivery systems, glucose-binding protein (GBP) systems, phenylboronic acid (PBA) systems, islet cell microencapsulation, nanotechnology-enabled closed-loop insulin delivery systems, and electrochemical and optical glucose measurement techniques. Balasubramaniyam et al. outline PLGA drug delivery systems, demonstrating their potential for reducing insulin injection requirements and improving patient treatment compliance and therapeutic success. The development of glucose-sensitive nanoparticle systems that mimic normal insulin pattern requirements has emerged as a significant advancement, bringing various potential solutions for better diabetes treatment [40]. Various nanoscale carbon structures are employed in electrochemical glucose monitoring because of their outstanding conductivity and catalytic characteristics. Electrical resistance variations can be monitored by connecting glucose oxidase to carbon nanostructures. Glucose concentration is determined by measuring enzyme activity at the electrode or watching changes in reactants or products. GOx may be immobilised on carbon nanotubes by forming amide bonds with carboxylic acid and amine groups. These nanotubes catalyse the H_2_O_2_ created during GOx activity, resulting in current flow proportional to glucose concentration, with a linear response in the range of 0–30 mM [39,41]. The approach for optical glucose readings is based on the enhanced fluorescence intensity generated by a fluorescent substance when paired with a glucose-sensitive material. Blood glucose levels are determined by altering the optical wavelength in response to glucose concentration. Carbon nanotubes are ideal for this use because they emit in the near-infrared spectrum, which is where the skin is most transparent [42]. Another innovation is the creation of closed-loop insulin administration devices, which constantly monitor blood glucose levels and automatically give insulin. This approach enables more accurate glucose management, which may reduce diabetes-related problems while also reducing the frequency of hypoglycaemia or hyperglycaemia episodes and overall insulin dose. Microencapsulation of islet cells is a step towards developing an artificial pancreas. Semi-permeable membranes surround β-cells, allowing insulin, nutrients, and tiny molecules to pass through while protecting against immune system assaults [43]. Designing such membranes is a challenging but critical endeavour. Glucose-binding protein (GBP) systems may attach to glucose units in glycopolymers, and lectins, notably Concanavalin A, are commonly utilised in insulin delivery systems. When blood glucose levels rise, G-insulin is released owing to the competitive binding of glucose and G-insulin. These systems frequently need external triggers, such as pH changes. Finally, insulin patches provide a non-invasive administration approach that has been widely accepted. However, previous attempts encountered difficulties such as limited absorption and the hostile environment of the gastrointestinal system. For example, a microneedle-based patch containing insulin-responsive nanovesicles was developed for minimally invasive insulin administration. While promising, this method requires additional refining to increase its efficiency and success. One useful tactic for fighting serious illnesses and infections is nanotechnology. Carbon nanoparticles are the most effective metallic and non-metallic nanoparticles for use in bioimaging, medication delivery, and sensing. Carbon nanomaterials (CNMs) are showing promise as a treatment for diabetes mellitus [44]. Nanoparticles and nanomaterials are increasingly explored for drug delivery, with engineering techniques aiming to target diseased cells and reduce adverse effects by targeting specific bodily regions or cells. Comparing nanoparticles to bigger molecules reveals several characteristics that enable improved pharmacological behaviour. There are several ways to synthesise different nanoparticles, including liposomes, micelles, and gold nanoshells. Depending on the desired purpose, the dimensions along with the forms of these particles can be altered throughout the synthesis procedure. During synthesis, nanoparticles can aggregate into larger particles, which, depending on their makeup, may increase or even decrease their cytotoxicity. In targeted drug delivery systems, reactive groups or molecules like antibodies can be added to the surface of nanoparticles to change their surface chemistry [45]. Nanoparticles are the fundamental constituents of nanotechnology. A few of the changes that can be achieved in nanoparticles are shown in Figure 3.

To further elaborate, the dimensions of nanoparticles range between 1 and 100 nm. The various categorisations of nanoparticles include carbon-based nanoparticles, organic nanoparticles, and inorganic nanoparticles. Likewise, carbon-based nanoparticles are categorised as carbon black, graphene, fullerene, carbon nanotubes, and carbon nanofibers [46]. Different types of nanoparticles, along with their characteristics, are tabulated below in Table 2.

**Figure 4:**
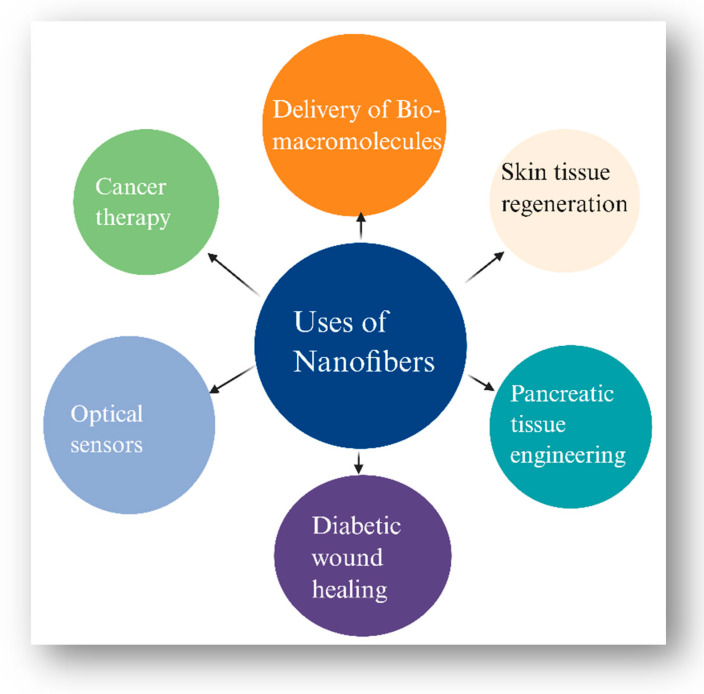
Applications of nanofibers in biomedical and therapeutic domains.

To continue with another class of nanomaterials, atomic sheets stacked in tubes that self-assemble are called nanotubes. Both organic and inorganic materials can be used to create them, and they can have one or more walls. In a common kind of nanotube, soluble fullerene derivatives like C60 are used. The exterior surface of nanotubes is easily functionalised, and their internal contents are vast. Although these compounds have the potential to be useful in pharmaceutical applications, there is conflicting information regarding their toxicity and human tolerance. The acute toxicity of nanotubes has been shown, and they may kill cells through an oxidative stress mechanism. The special qualities and uses of carbon nanotubes and carbon nanofibers in the realm of nanotechnology have earned a lot of interest [52,53]. CNFs have either a conical or cylindrical shape, and their diameter can range from a few to hundreds of nm [54]. Nanotubes made of carbon can be either multi-walled (MWCNT) or single-walled (SWCNT) [55,56]. Moving forward, in both industrialised and emerging nations, insulin-dependent diabetes mellitus is a multifactorial autoimmune illness that is becoming more and more common. Because of this, people with diabetes need to receive injectable insulin multiple times a day to control their blood sugar levels. A wide range of non-invasive delivery techniques is being thoroughly researched these days to lessen the discomfort and trauma connected with intravenous insulin administration. Investigating the potential of CNTs as a multipurpose insulin carrier could be intriguing. For instance, to facilitate the delivery of the attached insulin via the gastrointestinal tract, when taken orally, CNTs used as insulin carriers can be functionalised with mucoadhesive absorption enhancers or enzyme inhibitors [57,58]. Additionally, nanofibers are extremely fine fibres that can be created from polymer solutions and have diameters in the nanoscale [59]. It is essential to use alternative distribution methods to avoid restrictions or issues and to increase the efficacy and fulfilment of diabetes patients. Several distribution methods based on nanostructures were investigated to address various DM-related issues. Consequently, NF-based systems have demonstrated remarkable potential as delivery methods and as synthetic scaffolds for the administration of cells and medicinal substances [60,61,62]. A few uses of the NF-based system are illustrated in Figure 4 [63].

Delving deeper, diabetic wounds remain a substantial therapeutic concern due to a multifactorial poor healing process induced by peripheral neuropathy, diminished vascular function, impaired angiogenesis, and/or chronic inflammation, as well as microbial infection in chronic wounds. NF dressings and scaffolds are cutting-edge wound healing technologies that create ECM-like networks that can carry chemical and herbal medications, growth factors, and nanomaterials under control while also accelerating and promoting cell differentiation and proliferation [64,65]. NFs for the treatment of diabetes mellitus were made from a variety of biopolymers, which fall into two main categories: polysaccharides and polypeptides. Examples of these include collagen, gelatine (Gel), cellulose, chitosan (CS), hyaluronic acid (HA), silk fibroin (SF) of polypeptides, and alginate from polysaccharides [66]. The II–VI or III–V columns of the periodic table are commonly used to create quantum dots (QDs), which are semiconducting materials. At the molecular or bulk levels, they do not behave like semiconductors. The features they exhibit are dictated by their size, which falls between the range of 10 to 100 Å in radius [67]. With dimensions of nm, these semiconductor nanocrystals display quantum size effects in their electrical and optical characteristics [68]. The ability to synthesise QDs with a variety of surface substituents and sizes is one of their main advantages. Fluorescent labels with high quantum yields, narrow emission peaks, photobleaching resistance, and long luminescence lifetimes are promising compared to conventional fluorophores [69,70]. Like bulk semiconductors, QDs are crystalline materials with faces and a comparable lattice structure. Depending on its size, each nanocrystal can contain hundreds or thousands of atoms, with a considerable part (>10%) of those atoms present near the nanocrystal surface. Under ultraviolet (UV) light, differently sized QDs exhibit a vivid rainbow of photoluminescence (PL). Large molar extinction coefficients and high quantum yields combine to produce the brilliant PL [69].

## 4. Smart Nanocarriers for Precision Insulin Delivery

The integrated approach of nanotechnology and macromolecular research is being investigated as a novel way for insulin administration. Among these, systems based on nanoparticles have attracted notice. Usually between 100 and 1000 nm in size, polymeric nanoparticles are influenced by their size, surface charge, and molecular weight, all of which help determine how well they can transport insulin to the desired location. For enhanced insulin absorption and efficiency in diabetes control, these systems generally rely on two types of polymers: natural and synthetic. Natural polymers such as chitosan, alginate, dextran, and gelatine are being studied intensively for their safety and benefits. Chitosan, for instance, can be used orally and nasally; its qualities include biocompatibility, antibacterial, antifungal, and stickiness to mucous surfaces. It also lets one crosslink at many degrees. Chitosan-based nanoparticle formulations include reduced gold nanoparticles and chitosan-polyelectrolyte complex nanoparticles [71]. Mostly for oral administration, alginate is recognised for its capacity to create films and gels. It is also safe to use, biodegradable, and hydrophilic; it has been mixed with nanoparticles such as alginate-chitosan-cyclodextrin NPs, alginate-chitosan polyelectrolyte complex NPs, and alginate-chitosan coated NPs. Being biocompatible, having high solubility, and having many branching sites make dextran appropriate for oral administration systems, such as dextran-alginate sulphate nanoparticles with chitosan and albumin coatings [72]. Biocompatible as well, gelatine has a decent adhesive and soluble quality, creates gels well, and is suitable for oral and pulmonary applications. Systems such as gelatine–glutaraldehyde nanoparticles and gelatine-poloxamer nanoparticles take advantage of Polymers such as PLGA (poly-lactic-co-glycolic acid), polyvinyl alcohol (PVA), and polyamino acids are commonly employed on the synthetic side to enhance the delivery systems even further [73,74]. One can take PLGA orally, via injection, or intraperitoneally. Made from lactic and glycolic acid, it is a biodegradable, non-toxic polymer used in insulin delivery methods, including zinc-insulin-loaded PLGA nanoparticles, plain PLGA nanoparticles, and PLGA conjugated with chitosan nanoparticles. Biodegradable and great for transdermal and oral distribution, polyvinyl alcohol originates from the hydrolysis of polyvinyl acetate. It shows up in PVA nanoparticles as well as in insulin-loaded hydrogels produced from PVA and chitosan. Finally, polyamino acids are formed by polymerising amino acids or their derivatives. These are found in systems such as chitosan with poly-gamma-glutamic acid nanoparticles, gelatine-coated chitosan with poly-gamma-glutamic acid nanoparticles, and L-valine with poly(butyl acrylate) nanoparticles and are employed in oral administration [75,76,77]. Table 3 demonstrates the list of the natural and synthetic polymers employed in nanoparticle-based insulin delivery systems:

Alongside polymeric systems, lipid-based nanocarriers have surfaced as potent drug delivery vehicles, especially for oral routes. Lipid nanocarriers, including liposomes, nanostructured lipid carriers, and self-microemulsifying drug delivery systems (SMEDDS), provide biodegradability, high drug-loading capacities, scalability, and sustained release mechanisms. Composed of single or multiple phospholipid bilayers, nanoliposomes allow encapsulation of both hydrophilic and hydrophobic medications and permit cellular transport via endocytosis and micropinocytosis, hence enhancing drug stability and membrane permeability. Moreover, thermodynamically unstable nanoemulsions, kinetically stable dispersions of oil and water phases, sometimes > 100 nm in size, remain alive for weeks or months. Microemulsions are characterised as colloidal dispersions stabilised by high surfactant concentrations; they vary by providing improved thermodynamic stability. Usually falling between 20 and 200 nm, these systems generally cover a particle size spectrum of 10–1000 nm [78,79].

At the same time, microneedle arrays are transforming transdermal insulin administration. Vertically aligned conical microstructures tens to hundreds of microns in length gently penetrate the stratum corneum, generating microchannels that allow medication absorption. Many of which are made from polymers because of their changing physicochemical properties, affordability, biocompatibility, and manufacturing simplicity, designs feature hollow, solid, degradable, dissolving, and bio-responsive microneedles [80,81,82]. In recent years, polymers have received increasing interest in the field of microneedle fabrication because of their tunable physicochemical properties, improved biocompatibility, low cost, easy processability, and lack of sharp waste [83]. Microneedle arrays can be divided into either degradable or dissolving, based on how quickly the polymer dissolves. Dissolving microneedles consist of highly water-soluble polymers that dissolve within a very short time, resulting in a rapid release, while degradable microneedles are characterised by extended release profiles [84]. In situations involving diabetes, the external administration of insulin is crucial. Generally, patients administer insulin themselves through subcutaneous injections multiple times throughout the day. This process can involve discomfort, the potential for infection, and the risk of injury. Additionally, various soluble polymers can be used (such as HA [85], PVP [86], gelatin [87]), which contain insulin as a matrix, and it is combined with glycopolymers (e.g., Starch, gelatin, maltose, etc.). There are many advancements in drug delivery systems that mimic the response of the body to changes in blood glucose levels and release insulin as required. Polymeric microneedles loaded with insulin are designed with glucose-responsive mechanisms. When glucose levels fluctuate, the system allows for the release of the insulin payload via the dissolution or swelling of the microneedles. A nanoparticle with a range of 100–200 nm made of a hydrophilic polymer network called nanogel. Nanogel has a wide surface area, high stability, a sustained and targetable way, high drug loading capacity, and swellable and degradable features. Moreover, it serves as a valuable resource for developing groundbreaking medical treatment systems. These are pliable substances capable of containing inorganic nanoparticles, pharmaceuticals, and small molecular biomacromolecules within their interconnected networks. This allows them to be utilised for both visualisation and therapy of various medical issues [88]. These techniques have an advantage over conventional systems because they produce regulated medication release that is responsive to glucose. “Kataoka et al.” was the first to report that the amounts of insulin could be delivered based on the presence of glucose in glucose-responsive hydrogels containing phenylboronic acid groups [89]. A gel containing insulin that is formulated from auto-assembled nanoparticles of carboxymethyl-hexanoyl chitosan and includes the enzyme lysozyme for regulated biodegradation and insulin discharge serves as another example of self-regulated and controlled release [90].

## 5. Nanosensors in Glucose Monitoring

Diabetes is a chronic metabolic illness defined by a constant rise in blood sugar levels due to failure in insulin secretion and the absorption mechanism [91]. Patients with type 2 diabetes must strictly maintain their fasting glucose levels, which should be between 72 and 120 mg/dL and less than 153 mg/dL 1–2 h after meals [92]. Because invasive glucose detection technology is well-known, practical, and beneficial, both hospitals and home glucometers currently use blood collection first, followed by analysis to measure blood sugar in vitro. An automated biochemical analyser is used in hospitals to monitor blood glucose levels precisely after people’s blood is drawn in the morning, when they are not eating. This method’s results are accurate and can serve as a crucial foundation for the diagnosis of diabetes, but because of its time-consuming nature, lengthy diagnosis period, and high volume of venous blood, it is not appropriate for ongoing patient monitoring of people with diabetes [93]. Blood glucose monitoring involves tracking blood sugar levels using electronic home glucose metres, glucose oxidase biosensors, disposable paper tape, and chemical reaction streams [94]. However, most commercial glucometers still use pricey implanted devices or invasive sample procedures (such as venipuncture or finger prick) that are highly uncomfortable for frequent assessments [95]. Numerous fields of physiology and medical science have advanced significantly because of new techniques utilising nanotechnology. The technology is utilised for creating, constructing, characterising, and communicating tiny functional architectures in collaboration with biological science and engineering [96]. A biosensor typically comprises a detector, a signal transducer, and a biorecognition device. Initially, binding to or interacting with the analyte or process of interest is the recognition element, which might include antibodies, peptides, nucleic acids, or enzymes [97]. Nanomaterials have improved detection capabilities with great sensitivity and precision, and they also boost the surface-to-volume ratio [98].

In this regard, the significance of detecting glucose in bodily fluids for managing diabetes has led to the development of numerous glucose testing methods [99]. Wearable biomolecule sensing allows for the measurement, diagnosis, and uninterrupted monitoring of biofluid biomarkers such as electrolytes, metabolites, pH, vitamins, hormones, and immunodetections in patients [100]. Compared to other enzymes, the glucose oxidase (GO_x_) enzyme is the most widely used and the foundation of many glucose biosensors due to its exceptional selectivity for the glucose target, besides its ability to tolerate a wide range of pH and temperature settings [101]. Another important class of enzymes used in glucose measurement is glucose dehydrogenases (GDHs). The cofactors of these enzymes determine their classification. The cofactors for flavin adenine dinucleotide (FAD)- and pyrroloquinoline quinone (PQQ)-GDH are firmly attached to the enzyme, but the NAD cofactor is not structurally attached to the enzyme in nicotinamide adenine dinucleotide (NAD)-GDH. Although GDHs have the benefit of being O_2_-independent, GOx has higher specificity for glucose than FAD- and PQQ-GDHs [102]. First-generation glucose biosensors typically use GOx-catalysed glucose oxidation by the O_2_ natural substrate and measure the O_2_ cofactor’s depletion or the enzymatically produced H_2_O_2_ product. Therefore, by transporting electrons to the electrode surface, dissolved oxygen helps these sensors overcome the steric barrier of the FAD coenzyme. In addition to their potential for miniaturisation of both in vitro and in vivo clinical applications, first-generation glucose biosensors have the benefits of affordability and ease of use [103]. The second generation of glucose biosensors was recommended to utilise various co-substrates due to issues with oxygen dependence and electroactive interferences in the first generation. In place of O_2_, a synthetic electron acceptor is utilised, which transfers electrons from the enzyme’s buried redox-active site to the conducting electrode with rapid electrode kinetics and robust reversibility at low overpotentials [101]. Even though enzymatic sensors currently control most of the glucose sensor industry, there are still several issues that need to be resolved. These issues are mostly related to the intrinsic properties of the enzymes, including their low stability and limited repeatability after extended use. pH, temperature, humidity, and harmful substances can all readily affect an enzyme’s catalytic activity [104]. Despite these significant efforts, non-enzymatic glucose sensors have not yet made it to the commercial glucose market due to several issues that hinder their usefulness in the monitoring of diabetic patients. First, because they lack a selective recognition factor in their structure, they have low selectivity. Second, they frequently use alkaline solutions, which are very different from physiological pH levels. Third, rather than focusing on glucose detection specifically, the majority of investigations concentrated on the materialistic elements of the nanomaterials’ creation and construction. Future developments in nanomaterial synthesis and a better comprehension of the mechanisms underlying their catalytic pathways may enable nanomaterials to imitate the 3D architecture of enzymes for real-world uses, such as the glucose sensor sector [105]. Sweat and skin ISF are two easily accessible biofluids that have been used to study non-invasive epidermal electrochemical monitoring. These biofluids reflect blood glucose concentrations by diffusing glucose from blood arteries via the endothelium or sweat glands [106]. The integration of wireless electronics and body-compliant wearable platforms, such as patches and wristbands, can enable non-invasive glucose sensing through sweat or ISF. Although skin-worn glucose biosensors have great potential for bettering patient outcomes and managing diabetes, these systems still need to be developed further, critically assessed, and thoroughly validated before they can be widely used. A fresh strategy for tackling some of the most urgent issues in diabetes care is offered by these novel epidermal electrochemical devices [107]. The GlucoWatch^®^ biographer (Cygnus Inc., Ponderay, ID, USA) was the first non-invasive glucose monitor to be licenced by the FDA for commercial use in the United States. The glucose content of skin ISF collected by reverse iontophoresis (RI) was electrochemically evaluated by this wrist-worn device [108]. Ion migration across the skin is induced by providing a moderate current using two electrodes that are worn on the skin. The flux of positively charged sodium ions creates an electro-osmotic flow towards the cathode because of the skin’s negative charge, which also causes the neutral glucose to travel towards the same electrode [107]. Due to its many benefits, including the greatest number of sampling sites outside the body, continuous access, ease of placement and comfort of collection devices, and its composition of physiologically significant electrolytes and metabolites, sweat is a very appealing biofluid for non-invasive, continuous monitoring applications [109]. A few examples of wearable biosensors are GlucoWatch^®^ biographers and temporary tattoos; these sense glucose from ISF [110,111] using reverse iontophoresis. Other advanced examples include multiplexed, wearable, flexible array patches; sensor array patches coupled with induced sweating; graphene-based stretchable patches; and multimodal wearable patches, which sense glucose from sweat, ISF, or tear fluid depending on the sensor design and transduction mechanism [112,113,114,115].

In addition to wearable devices, implantable nanosensors also provide promising solutions for long-term glucose monitoring. Implanted nanosensors may be stationary at an anatomic location or circulate in the circulation. To report analyte data, they can theoretically generate electrical, optical, magnetic, or audio signals. Although circulating nanosensor designs are fascinating, they also bring up several issues that are covered elsewhere and are too comprehensive for this study, including biodistribution, circulation lifespan, and clearance [116]. The Eversense CGM System, a long-term implantable subcutaneous tissue CGM device made by Senseonics, Inc. (Germantown, MD, USA), measures the interstitial fluid glucose concentration every five minutes and shows the results on the patient’s cell phone for 90 days in the US or 180 days in Europe and South Africa. The Eversense System has an accuracy comparable to other commercial systems for measuring glucose concentrations between 40 and 400 mg/dL. However, to precisely detect the concentration of interstitial tissue fluid glucose, MARD (mean absolute relative difference) 8.5% to 11.5%, the implanted sensor needs to be recalibrated using a finger stick SMBG (self-monitoring blood glucose) measurement roughly every 12 h. When making decisions about diabetes treatment, the CGM system is intended to take the place of fingerstick blood glucose measurement [117]. The Minimed Continuous Glucose Monitoring System was the first FDA-approved CGM sensor, featuring an implantable needle-style sensor, transmitter, and display monitor. Calibration was required daily for the original sensors. Even though CGMS’s composition has not changed much, the Abbott FreeStyle Libre and Dexcom G6^®^ CGMSs that are now in use no longer need to be calibrated. Since sensor manufacturing advancements have led to a fundamental transformation in CGMS, Dexcom G6^®^ and Abbott FreeStyle Libre can provide zero-finger pricking glucose monitoring without the need for in vivo calibration [118]. Furthermore, optical sensing technology, based on photon variations in light characteristics, is a potential candidate for CGM biosensors due to its sensitivity and adaptability [119,120,121]. The following kinds of optical biosensors can be distinguished based on transducer systems: fluorescence; in the study, various biosensors are explored, including Raman, SPR, near-infrared, optical coherence tomography, Fourier transform near-infrared, and surface-enhanced Raman scattering biosensors [121].

**Figure 5:**
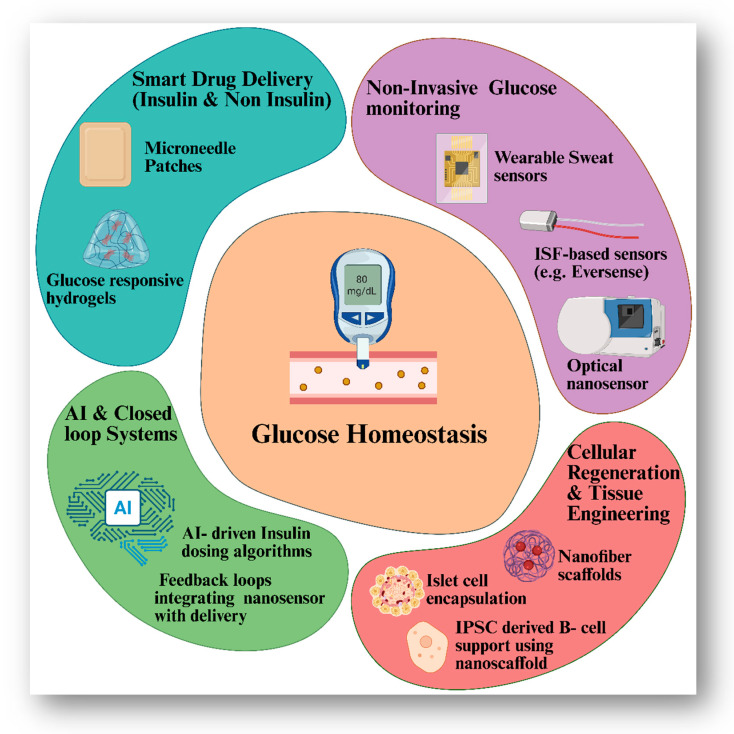
Synergistic nanotech framework for advanced diabetes management.

As illustrated in Figure 5, nanotechnology enables a comprehensive and interconnected approach to diabetes management, combining real-time biosensing, smart insulin delivery, regenerative tissue engineering, and AI-guided decision systems. In addition to monitoring, nanotechnology also contributes to the regenerative aspect of diabetic therapy. The therapeutic management of diabetes mellitus and its numerous consequences has advanced recently because of the growth of diabetic regenerative medicine. Additionally, the development of nanotechnology has given diabetic regenerative therapy fresh life. Nanostents can effectively guide the regeneration of islet β cells, nerve tissue, wound tissue cells and retinal tissue. Conductive nanoparticles promote the development of many tissues [122]. An effective long-term treatment for type 1 diabetes that does not require compliance and does not cause complications is islet transplantation. However, there are some disadvantages, such as a scarcity of donor islets, immunosuppressive side effects, etc. So, islet transplantation is now accessible only to a small number of patients [123]. Endogenous regeneration induction and transplanting of cadaver islets, as well as β cells produced from human embryonic stem (hESC)/inducible pluripotent stem (iPSC) cells, are two techniques for supplementing β cells due to islet β cell dysfunction. To put it another way, it happens because of progenitor differentiation (neogenesis) or transdifferentiation. In the meantime, research on pluripotent stem cells or inducible pluripotent stem cells (iPSC), allogeneic islets, particularly pig islets, has been prompted by immunological rejection and a lack of islet organ donors [124,125,126]. A scaffold matrix is necessary to support the pancreatic tissue’s 3D development. To correct hyperglycaemia in diabetic mice, for instance, islet-like cells generated from human embryonic stem cells were seeded onto poly(lactic-co-glycolic acid) scaffolds [125].

## 6. Peptides in the Treatment of Diabetes

The rapidly increasing global incidence of diabetes mellitus, especially type 2 diabetes mellitus (T2DM), calls for more efficient, specific, and patient-friendly treatments. Among the encouraging ideas are peptide-based therapies, which have advanced considerably in the last century. Beginning their therapeutic path with insulin extraction from canine pancreatic tissue in 1921, peptides in diabetes were first clinically used in 1922. This groundbreaking work, spearheaded by Frederick Banting and Charles Best, set the basis for current diabetes therapy. The 1982 creation of recombinant human insulin via recombinant DNA technology ushered in the switch from biosynthetic to animal-based products. This suggested a significant rise in supply networks and safety. In the years that followed, studies focused mostly on enhancing insulin pharmacokinetics. Rapid-acting analogues like Lispro (Humalog^®^), long-acting versions like Glargine (Lantus^®^) and Detemir (Levemir^®^), and ultra-long-acting forms like Degludec [127] made it possible to have more flexible, patient-tailored regimens that helped to lower glycaemic variability and the risk of hypoglycaemia, as well as to enhance adherence and results. Incretins, a class of hormones produced by the intestines, attracted notice at the same time as insulin. The “incretin effect” describes the phenomenon whereby intestinal L-cells secrete glucagon-like peptide-1 (GLP-1) and glucose-dependent insulinotropic polypeptide (GIP), respectively, resulting in insulin secretion that is significantly higher after oral glucose intake compared to intravenous administration [128,129]. In a glucose-dependent way, GLP-1 increases insulin release, decreases hunger, slows stomach emptying, and inhibits glucagon release. More recent clinical studies have found supporting evidence validating the use of GLP-1 receptor agonists regarding glycaemic control and protection against cardiovascular diseases. For example, in the REWIND study, where diabetes participants were administered dulaglutide, there was a remarkable reduction in major adverse cardiovascular events (MACE) among T2DM patients with high cardiovascular risk [130]. Also, the LEADER study with liraglutide reported a 13% decrease in cardiovascular risk in comparison to the placebo group [131]. In addition, SUR-PASS trials suggest tirzepatide, a dual GIP/GLP-1 receptor agonist, shows greater reduction in HbA1c and body weight when compared to semaglutide and other typical GLP-1 receptor agonists [132]. Although GLP-1 has certain advantages, the enzyme dipeptidyl peptidase-IV (DPP-IV) swiftly breaks it down; hence, it is not often used in medicine. In response to this difficulty, GLP-1 receptor agonists (GLP-1 RAs) resistant to DPP-IV degradation were developed. The first oral GLP-1 RA to hit the market was semaglutide; subsequent treatments, including Liraglutide, Dulaglutide, and Exenatide, followed suit following its 2005 approval. In terms of lowering HbA1c levels, encouraging weight loss, and safeguarding the heart, these medications have been beneficial [131,133]. A major development in incretin therapy was the introduction of tirzepatide, a dual GIP/GLP-1 receptor agonist. Compared to present GLP-1 RAs, tirzepatide demonstrated better glycaemic control and weight loss results in the 2022 SURPASS trial series. Off-label research with GLP-1 RAs as adjuvant therapy has taken place in type 1 diabetes, a condition characterised by lifetime insulin dependence brought on by the autoimmune destruction of beta-cells. In real-world studies including teenagers and young adults, semaglutide and tirzepatide increased glycaemic levels, reduced insulin needs, and encouraged weight reduction [134]. Because these medicines may have severe ramifications, it is important to appropriately monitor side effects and psychological repercussions, including disordered eating. Traditionally regarded as a physiologically inactive byproduct of insulin production, C-peptide is rather crucial for diabetes control. A strong marker for evaluating endogenous insulin release is C-peptide, which is produced in equimolar levels with insulin, since it resists hepatic metabolism and has a long half-life. C-peptide testing in clinical practice helps to identify diabetes subtypes, forecast how a patient will respond to treatment, and make plans for when and how to stop taking insulin. While high or preserved C-peptide levels indicate the possibility of non-insulin treatments, low levels may indicate early insulin commencement [135]. According to research, C-peptide, which was previously thought to be physiologically inert, may help treat type 1 diabetes mellitus. C-peptide may also improve microvascular blood flow, nerve transmission speed, and kidney function. According to a phase II trial by Johansson et al., C-peptide therapy improved peripheral nerve function in individuals with T1DM and early-stage neuropathy. However, a more thorough study is required to validate its long-lasting effects and safety [136,137]. While clinical practice has been mostly focused on hormone-based peptide treatments, bioactive peptides derived from food can be a complementary or maybe replacement therapeutic option. Released by the enzymatic degradation of dietary proteins, these peptides range in length from two to twenty amino acids. Among the antidiabetic actions shown by plant-derived peptides are suppression of alpha-amylase, alpha-glucosidase, and DPP-IV enzymes, as well as activation of insulin signalling pathways such as PI3K/Akt and MAPK [138]. Peptides that imitate the effect of insulin, increase insulin production, and lower postprandial glucose spikes have been found in foods including bitter melon, soy, black beans, wheat, and peas. For example, oat protein peptides increase insulin sensitivity and decrease bodily inflammation, hence helping to control overall metabolism. Natural DPP-IV inhibitors originating from dairy products, especially hydrolysed whey protein, have also recently come to light. Camel milk peptides (VPV), alpha-lactalbumin peptide (LDQWLCEKL), and other beta-casein or serum albumin peptides exhibit DPP-IV inhibition at IC50 values similar to pharmaceutical gliptins [128]. Intended for medical nutrition therapy and functional foods, these peptides maintain incretin activity and show anti-inflammatory, satiety-promoting, and antioxidant properties as well. Their all-natural composition and low risk of negative effects make them more easily included in regular meals than produced medications. Although peptides have great potential, clinical use is restricted because of issues including low oral bioavailability, fast disintegration, and short half-lives. Modern pharmaceutical researchers have tackled these issues with ways to boost the stability and absorption of peptides, including encapsulation in nanocarriers, liposomes, or hydrogels, structural changes, and PEGylation [139]. One such use of an absorption booster (SNAC) is the formulation of oral semaglutide. This enhancer changes the stomach’s pH temporarily and prevents enzymes from breaking down the peptide. Since they require fewer injections and have better adherence rates, these developments pave the way for peptide treatments that are more patient-friendly. Peptide treatments in diabetes have bright future possibilities. From insulin injections, which have saved countless lives, to innovative dual agonists and nutraceutical peptides, these substances are always redefining the possibilities in diabetes control. Important actors in full, individualised therapy have many effects, including glycaemic management, weight loss, anti-inflammation, β-cell maintenance, and cardiovascular protection. Peptide engineering, delivery technologies, and food-based functional interventions are all evolving, so, future diabetic treatments will likely be hybrid approaches combining synthetic precision with natural bioactivity.

## 7. AI-Driven Glucose Monitoring

AI has been developing over the last ten years to enhance CGM biosensor performance. The American Diabetes Association advises using AI to detect diabetic macular oedema and moderate diabetic retinopathy, in addition to using it as a substitute for conventional screening methods. AI techniques such as support vector machines, artificial neural networks, supervised machine learning, and principal component analysis methods are widely used in diabetes treatment. In several diabetes care application scenarios, including calibration, decision support systems, closed-loop control, patient self-management tools, and automated retinal screening, the integration of machine learning with CGM has demonstrated potential [140,141,142]. A person with type 1 diabetes requires insulin therapy and must frequently adjust their dosage to meet their glycaemic goals. Closed-loop control systems, also known as artificial pancreases, can automate insulin therapy for diabetes treatment. The first commercially available artificial pancreas device with full integration, the MiniMed 670 G, was approved for the treatment of type 1 diabetes in 2016 [143,144,145]. An essential component of closed-loop systems is the control algorithm. It can be found in smartphones and integrated into insulin pumps [143]. A system that uses information from CGM biosensors to give people specific advice is called a decision support system [146]. Compared to the autoregressive model, the ANN method showed better accuracy in real-time estimation utilising CGM biosensor data, with a smaller root mean square error [147].

## 8. Critical Perspective on Clinical Translation, Safety, and Efficacy of Nanotechnologies in Diabetes

Clinical application of nanotechnology in diabetes is still limited, despite impressive preclinical advancements. Many promising nanosystems have demonstrated high efficacy in lab or animal studies, but they face obstacles in human application. Examples include glucose-responsive microneedles, nanogel-based insulin delivery, and quantum dot glucose sensors. Safety continues to be the top priority. Depending on the particle size, shape, surface charge, and dosage, nanomaterials like carbon nanotubes, metal nanoparticles (such as silver or gold), and quantum dots may be cytotoxic, genotoxic, or immunogenic. Prolonged buildup in organs like the kidneys, spleen, and liver can cause inflammation or oxidative stress [148,149]. For example, research on carbon-based nanomaterials shows that when they are used without surface modifications, they cause mitochondrial disruption and oxidative damage in pancreatic cells [150]. Standardised regulations for nanoformulations are also still being developed. Different labs produce different results because there are no standardised safety assessment procedures. Few clinical studies have been conducted; only a small number of FDA-approved nano-based glucose sensors (such as GlucoWatch and Eversense) require invasive implantation or frequent calibration [151,152]. High manufacturing costs, batch-to-batch variability, and the limited scalability of lab-synthesised nanoparticles are further examples of translational barriers. Clinical-grade manufacturing of lipid-based systems and polymeric nanoparticles is challenging due to their propensity for instability during storage [153]. In vivo performance is further hampered by immune clearance and the unpredictability of nanoparticle biodistribution. Clinical efficacy is an additional difficulty. Not many studies show long-term glycaemic control or a decrease in diabetes-related complications in human populations, even though some polymeric nanocarriers (such as those based on chitosan or PLGA) increase insulin bioavailability. Most clinical studies continue to be in phase I or II. To get around these problems, future research should focus on improving biocompatibility by changing the surface (for example, by PEGylation), utilising intelligent materials that respond to physiological cues like pH or glucose, conducting thorough preclinical research using standard methods, and planning clinical trials that consider clinical endpoints like HbA1c, patient-reported outcomes, and adverse events in addition to pharmacokinetics.

## 9. Future Directions and Emerging Trends

Researchers and innovators have contributed a lot to diabetes and various diabetic drug delivery systems till today. Subcutaneous injection is the conventional approach for delivering insulin can be administered using several methods, including insulin syringes, insulin infusion pumps, oral hypoglycaemic medications (chemical agents), insulin infusion pumps, and jet injectors. A major challenge with existing insulin treatments is that they are invasive. Effective blood sugar management in type 1 diabetes mellitus is accomplished through two or more insulin injections each day. This continuously evolving field requires ongoing innovation to advance technology and address future challenges effectively. Here is a brief of some of the modern insulin delivery methods [154]. Insulin pen injectors represent significant improvements in insulin delivery, making self-injection more convenient and easier. A significant advancement in insulin drug delivery systems is achieved using external insulin pumps, insulin inhalers, and implantable insulin pumps. Insulin inhalers were removed from the market in October 2007 because of worries regarding lung cancer. Nevertheless, a novel inhalable insulin product obtained FDA approval for distribution in the US in June 2014. In contrast to traditional subcutaneous insulin, inhaled insulin produced quick and long-lasting patient satisfaction as well as a favourable effect on psychological health in individuals with type 1 diabetes [155]. However, inhaled insulin is more quickly absorbed, the duration of action is short, the bioavailability of inhaled insulin is very low, and the cost per dose is higher compared to subcutaneous injection. The use of insulin pills is also one of the emerging trends. Since insulin is a protein, it must be protected from the stomach environment and enzymes that break it down to be delivered accurately. One of the primary considerations in the development of an oral insulin medication is the need to safeguard against degradation. Polymers should be utilised for this protection. For instance, Azopolymer can facilitate delivery to the colon area. Alginate can be used to make pH-responsive insulin delivery systems [156]. Innovative Novel Drug Delivery technologies are essential for this endeavour [154]. Islet Cell Transplant is also one of the new emerging trends. It aims at maintaining a constant normoglycaemic state and avoiding hypoglycaemic episodes [157]. The use of insulin analogues has also gained popularity. So, an insulin analogue mimics natural insulin present in our body. Instant-acting insulin analogues include “Aspart, Glulisine, Lyspro”. Long-acting injected insulin analogues include insulin glargine and insulin detemir. There are some premixed analogues in the market that contain natural insulin and insulin analogues in fixed proportions [158]. As nanotechnology advances, the applications in diabetes control will become more realistic and transformational. Nanomedicine has the potential to transform the future of diabetes treatment by bridging existing research gaps and overcoming translational hurdles, enabling more precise, tailored, and effective solutions for preventing and treating diabetic problems.

## 10. Conclusions

To sum up, nanotechnology has emerged as a novel, promising area in the treatment of diabetes. Researchers have made innovative strategies to address the complex problems along with the chronic illness, taking into consideration the special properties of nanomaterials. Nanotechnology can radically change the treatment of diabetes, especially through innovative medication delivery systems and sophisticated sensing technologies. Many nanotechnological strategies have thus been discussed within this review, including the making of implantable devices, glucose-reactive materials, and nanoscale drug carriers. These can improve patient compliance, lower the adverse effects, and enhance the therapeutic efficacy. In addition, nanotechnology-powered sensing technologies allow monitoring the glucose levels in real-time, which enables immediate intervention and individualised treatment regimens. Much has been achieved so far, but much more still lies ahead in terms of R&D to bring such promising science to practical clinical uses. The critical biocompatibility, toxicity, and scalability issues still need to be overcome. Nevertheless, good times await in diabetes management as nanotechnology promises to help millions suffering from this debilitating disease.

## Figures and Tables

**Table 1:** Dose, effect of food, and side effects of current diabetes medications.

Category	Drug	Dose	Food Effect	Side Effect	Reference
Glinides	Repaglinide	0.6–4 mg	Take 30 min before meals	Drug interactions, upper respiratory infections.	[23]
Sulfonylureas	Glibenclamide	1.25–20 mg/day	Taken with food	Increased body weight, hypoglycaemia	[25,26,27]
	Glipizide	2.5–40 mg/day	30 min before meals		
	Gliclazide	Max 15 mg/day	Taken with food		
Dipeptidyl peptidase-4 inhibitors	Sitagliptin	100 mg daily	Not affected	Angioedema, pancreatitis	[28]
	Saxagliptin	2.5–5 mg	Not affected		
	Vildagliptin	50 mg BID	Not affected		
Biguanides	Metformin (IR)	500 mg BID, Max 3 g	During or after meals	GI disorders, vitamin B12 deficiency, lactic acidosis	[29,30]
	Metformin (XR)	750 mg OD, Max 2 g	With evening meals		
Thiazolidinediones	Pioglitazone	15–45 mg once/day	Not affected	Increased Body weight, anaemia	[31]
	Acarbose	25–100 mg	At the start of every meal	Hypoglycaemia, URTI	[32,33]
Sodium–glucose cotransporter-2 inhibitors	Dapagliflozin	10 mg OD	Irrespective of food	DKA, UTI, hypovolemia	[25,34]
	Empagliflozin	10–25 mg OD	Irrespective of food		

**Table 2:** Types of nanoparticles.

Nanoparticle Type	Properties	References
Liposomes	Lipid bilayer-encapsulated spherical structures are designed to protect medications from deterioration. They are effortlessly functionalised with targeted segments for a particular delivery and are biocompatible.	[47]
Dendrimers	Potentially useful as drug loading systems, these nanoscale polymers are monodispersed and highly branched. They come in a range of sizes, surface qualities, and drug-delivery capacities, making them highly customisable.	[48]
Polymeric NPs	Made from biodegradable polymers that can shield medications from deterioration by encasing them. They are frequently employed for the long-term, sustained delivery of medications.	[49]
Metal NPs	They are appealing for application in drug delivery because of their distinct optical, electrical, and thermal characteristics, such as those of gold nanoparticles (AuNP) and silver nanoparticles (AgNP). Targeting moieties can functionalise them for a particular delivery method.	[50]
Solid Lipid NPs	They are used for packing hydrophobic medications and are composed of solid lipids. Compared to other kinds of nanoparticles, they have several benefits, such as increased bioavailability, stability, and biocompatibility.	[51]

**Table 3:** Natural and Synthetic Polymers Used in Nanoparticle-Based Insulin Delivery Systems.

Polymer Type	Polymer Name	Route of Administration	Key Properties	Examples of Nanoparticles	Reference
Natural Polymer	Chitosan	Oral, Nasal	Biocompatibility, antibacterial and antifungal activity, mucoadhesiveness, varying degrees of crosslinking	Reduced gold nanoparticle systems; chitosan polyelectrolyte complex-based nanoparticles	[71]
	Alginate	Oral	Film-forming ability, gelling capability, hydrophilicity, biodegradability, non-toxicity, ionic crosslinking	Alginate–chitosan–beta-cyclodextrin nanoparticles; alginate–chitosan polyelectrolyte complex nanoparticles; alginate–chitosan-coated nanoparticles	[72]
	Dextran	Oral	High branching potential, water solubility, biocompatibility	Dextran–alginate sulphate nanoparticles with chitosan and albumin coating	[73]
	Gelatin	Oral, Pulmonary	Biocompatibility, gel-forming ability, adhesiveness, water solubility	Gelatine–glutaraldehyde nanoparticles; gelatine–poloxamer-based nanoparticles	[74]
Synthetic Polymer	Poly (Lactic-co-Glycolic Acid) (PLGA)	Oral, Intraperitoneal, Injectable	Biodegradable copolymer of lactic and glycolic acid; good biocompatibility; non-toxicity; plasticity	zinc–insulin-loaded PLGA nanoparticles; PLGA nanoparticles; PLGA–chitosan conjugated nanoparticles	[75]
	Polyvinyl Alcohol	Transdermal, Oral	Derived from the hydrolysis of polyvinyl acetate; biodegradable by microorganisms	Polyvinyl alcohol–chitosan hydrogel loaded with insulin; polyvinyl alcohol nanoparticles	[76]
	Polyamino Acids	Oral	Obtained by polymerisation of amino acids or derivatives; customisable monomeric structure; high compatibility	Chitosan and poly-gamma-glutamic acid nanoparticles; gelatine-coated chitosan/poly-gamma-glutamic acid nanoparticles; L-valine/poly(butyl acrylate) nanoparticles	[77]

## Abbreviations

AMPK	AMP-activated protein kinase
CGM	Continuous Glucose Monitoring
CNF	carbon nanofiber
CNMs	carbon nanomaterials
CNT	carbon nanotubes
DPP-4i	dipeptidyl peptidase-4 inhibitors
DM	diabetes mellitus
FRET	Förster Resonance Energy Transfer
GBP	glucose-binding protein
GDH	glucose dehydrogenase
GI	gastrointestinal
GIP	glucose-dependent insulinotropic polypeptide
GLP-1	glucagon-like peptide-1
GOx	glucose oxidase
HA	hyaluronic acid
IDDM	insulin-dependent diabetes mellitus
ISF	interstitial fluid
iPSC	inducible pluripotent stem cells
MARD	mean absolute relative difference
MWCNT	multi-walled carbon nanotubes
NIDDM	non-insulin-dependent diabetes mellitus
PBA	phenylboronic acid
PL	photoluminescence
PLGA	poly (lactic-co-glycolic acid)
PVA	polyvinyl alcohol
QD	quantum dot
SF	silk fibroin
SMBG	self-monitoring blood glucose
SGLT-2i	sodium–glucose cotransporter-2 inhibitors
SU	sulphonyl urea
SWCNT	single-walled carbon nanotubes
T1DM	Type 1 diabetes mellitus
T2DM	Type 2 diabetes mellitus
TZD	thiazolidinedione
